# Group music therapy for the proactive management of stress and anxiety

**DOI:** 10.1371/journal.pmen.0000312

**Published:** 2025-08-14

**Authors:** Rachael Finnerty, Laurel Trainor

**Affiliations:** 1 Department of Psychology, Neuroscience and Behaviour, McMaster University, Hamilton, Ontario, Canada; 2 McMaster Institute for Music and the Mind, Hamilton, Ontario, Canada; Hamdard University - Islamabad Campus, PAKISTAN

## Abstract

There are limited supports available on Canadian campuses for individuals who are motivated to proactively manage their mental health in comparison to the supports available to students seeking supports for a crisis. Research typically evaluates mental health treatments for individuals with a diagnosis, leaving a paucity of research examining the benefits of therapy for individuals not experiencing a mental health crisis. We conducted this randomized controlled study to explore the effectiveness of group music therapy as a tool for students to proactively reduce their stress and anxiety. Interested students were randomly assigned to either six-weeks of weekly group music therapy or a campus-life-as-usual control group. Significant reductions in cortisol levels from hair samples, as well as psychometric measures of stress and anxiety were observed in the music therapy group in comparison to the control group. Further studies are needed with specific control group comparisons to determine the aspects of group music therapy that led to stress and anxiety reduction. At the end of the study 71% of students responded that they found group music therapy helpful, and 66% responded that they would continue to participate in group music therapy if offered on campus. Additionally, 61% of the students in the control group indicated that they would like to attend group music therapy. These results support the inclusion of group music therapy as an option on university campuses for students interested in proactively managing their stress and anxiety.

## Introduction

Advocating for proactive mental health interventions on university campuses is not a new concept, yet services remain predominantly reactive to mental health concerns. The Ontario Undergraduate Student Alliance (OUSA) 2012 report included the recommendation of anti-stigma and preventative mental health campaigns as well as workshops for students at post-secondary levels to enhance coping and stress management [1]. Six years later, in the 2018 OUSA report, students identified a lack of proactive care and crisis prevention on campuses [2], and a scoping review of post-secondary stress and mental well-being highlighted the need for prevention strategies [3]. In 2022, an online survey delivered to post-secondary students across Ontario, Canada, revealed that 66.5% of respondents (*n* = 290/436) reported a decline in their mental health since starting post-secondary education [4]. Despite these mental health concerns, most students reported not receiving counselling or psychotherapy for their mental health (71.5%; *n* = 318/445). Some changes have been implemented since the 2012 OUSA report, but proactive engagement in mental health support is still not a part of campus culture, with mental health services predominantly for students already in crisis or with a specific diagnosis [5].

The present research explored the effectiveness of group music therapy to proactively manage undergraduate students’ stress and anxiety under conditions where participants did not require a diagnosis, measuring both physiological and psychological measures. Group music therapy was chosen as a more cost-effective and feasible option compared to individual music therapy. Group therapy also provides the opportunity to recognize similarities between one’s own experiences and those of others, hence validating and normalizing experiences [6]. Music is typically considered to be a healthy activity, without a link to mental illness [7]. Recognizing that many students do not seek mental health supports when needed due to negative stigma [2,4], music therapy could offer an alternative to often stigmatized verbal therapies, increasing mental health support-seeking. Additionally, music therapy has a higher participation and retention rate over verbal based therapies [8–11]. It should be noted that participants do not require any background in music to participate in or benefit from music therapy.

Music therapists work with a diversity of age groups and diagnoses using music purposely within a therapeutic relationship to support a variety of healthcare goals. Engaging in music therapy has led to improvements in verbal fluency, reduced anxiety, reduced symptoms of depression, reduced pain perception, improved psychosocial functioning, and improved motivation for treatment [12–17]. However, systematic reviews have also reported a need for clinical trials with larger sample sizes, appropriate experimental methodology, and objective measurements of treatment effectiveness to substantiate these claims [12–17].

The present randomized controlled trial analyzed the effects of undergraduate university students’ proactive participation in group music therapy for stress and anxiety management. In a previous paper, our group showed that online group music therapy could proactively manage stress and anxiety in undergraduate university students during COVID-19 restrictions [8]. The present study followed a very similar protocol, with the main difference being that the music therapy groups were delivered in-person on campus. In keeping with the online study, the music therapy groups followed a community music therapy approach, which does not require an intake form, an assessment, or a treatment plan. Additionally, this approach aims to effect change both within the therapeutic space and the overall environment of the client [18]. For instance, the present study aimed to assist with the management of stress and anxiety within the therapeutic space, as well as address the need to normalize the maintenance of mental health.

In recognition of the literature already supporting the outcomes of music therapy in comparison to the standard of care (verbal based therapy) [8,9,19–23], a standard of care group was not part of the present study design. Rather, stress and anxiety measures were compared between students who proactively participated in a music therapy group and students who experienced ‘student life as usual’ (the control group). Stress measures included cortisol (analyzed from hair samples), heart rate variability (HRV), the standardized perceived stress scale (PSS), and a 1-5 scale of self-perceived stress; and anxiety was measured using the standardized state trait anxiety inventory (STAI-S). The state version of the STAI-S was implemented as we were interested in changes in participants’ anxiety in the moment as opposed to participants predisposition to anxiety (trait anxiety). Cortisol was collected as a known biomarker for stress [24]. Collecting hair cortisol provides a proxy for total hypothalamic pituitary adrenal axis activity in the preceding months; each centimeter of hair growth reflects the average cortisol released in the previous month [25].

In the present study, we wanted to compare participants’ average cortisol release for the month before the study to their average cortisol release during the last month of the study, we therefore collected the first centimeter of hair growth from the scalp at the beginning and at the end of the six-week intervention for analyses. Heart rate variability was collected as an additional non-invasive measure of the autonomic nervous system that is recognized as a reliable assessment of stress [26], with increased variability in heart rate being associated with a greater ability to cope with uncertain environments [27]. The standardized perceived stress scale is designed to assess feelings about life events and situations over the previous months. This enabled the comparison of perceived stress before the study to after the study. The 1–5 scale of self-perceived stress provided a quick, in the moment, subjective experience of stress. In addition to collecting measures in week 1 and 6 of the study, the STAI-S 1–5 stress scale and HRV measures were also collected before and after each group music therapy session. These data provided information about the immediate effects of music therapy on stress and anxiety. This is important as experiencing low levels of stress, even for a short period of time, can have benefits [28].

Recognizing that demographic variables, personality traits, and extenuating circumstances could influence the stress and anxiety scores of students during the six-week study period, a Demographics questionnaire, the Ten Item Personality Inventory, and the World Health Organization – Quality of Life questionnaire were administered. Personality type can predict propensity to manage stress and anxiety, but there are only a few published research papers addressing the extent to which personality type affects the degree to which stress or anxiety scores change during a therapeutic intervention such as music therapy [29–31]. Participants also completed the Goldsmith Music Sophistication Index, which includes musical skills, expertise, achievements, and related behaviors [32], to determine if those with higher music sophistication would benefit more from music therapy. Despite these variables not being explicitly measured in previous music therapy research, we hypothesized that neither personality type nor music sophistication would be related to therapy outcomes, based on the diversity of individuals who reportedly benefit from music therapy, and music sophistication not being a requirement of participating in music therapy.

Participants’ subjective experiences are often not accorded the same weight as intervention outcomes, yet they are invaluable in understanding if individuals will seek out and/or continue to engage in supports [33]. For this reason, the present study included a feedback questionnaire for participants in the music therapy group, consisting of both closed- and open-ended questions about their experience and their interest in continuing in the intervention if it was offered.

In summary, the present research gathered behavioral and physiological measures of stress and anxiety, as well as data about potential moderating variables (demographics, personality traits, music sophistication, quality of life), and asked two main questions, and two secondary questions, as follows.

Two main questions:

(1) *Does participating in a 45-minute group music therapy session reduce stress and anxiety from before to after the session?*
(2) *Does participating in six weeks of weekly group music therapy reduce stress and anxiety in comparison to a “student life as usual” control group?*

Two secondary questions:

(1) *Do participants in music therapy report finding the intervention helpful?*
(2) *Would participants engage in music therapy if it was offered on campus?*

We hypothesized that a reduction in stress and anxiety would be observed both before and after each music therapy session (as measured by STAI-S, self-rating scale, HRV), as well as from week 1 to week 6 of the study (STAI-S, self-rating scale, HRV, PSS, cortisol). We also anticipated that participants would find the music therapy groups helpful, and that most participants would be interested in continuing to engage in music therapy if it was offered on campus.

## Materials and methods

### Overall study design

A randomized controlled trial, pretest-posttest study design with two groups was approved by the Hamilton Integrated Research Ethics Board (project #15143). The two groups consisted of an experimental group (music therapy) and a control group (student life as usual). University students were randomly assigned to participate in a 45-minute group music therapy session every week for six weeks, or to the control group. Demographic data, including a personality questionnaire and a music sophistication questionnaire were collected at the start of the study. A quality of life questionnaire was distributed in week 1 and week 6 of the study. Measures of stress and anxiety were taken before and after each group music therapy session, and in week 1 and week 6 of the study from all participants (see details below). Participants in the music therapy group were asked to complete a feedback survey at the end of the study.

### Participants

A total of 148 full-time undergraduate university students (80% females) aged 18–24 (M = 20 years) at a Canadian university participated in the study. A power analysis was conducted using G*Power version 3.1 [34] to determine the sample size required to test whether the average change in the state anxiety and self-rated stress scores reduced from before to after each group music therapy session, with 80% power for detecting a medium effect (*d* = 0.5), at a significance criterion of α = .05. These parameters were determined by referencing a similar study [8]. The sample size determined for this within group one-tailed paired *t*-test was **n* *= 27.

A power analysis was also conducted to determine the sample size required to test whether the average change in stress and anxiety scores from week 1 to week 6 differed between the music therapy group and the control group with 80% power for detecting a medium effect (*d* = 0.5), at a significance criterion of α = .05. These parameters were determined by referencing a similar study [8]. The sample size determined for this between group one-tailed t-test was *n* = 102 (51 participants per group).

In block 1 of the present study, recruitment was conducted January 10, 2023-February 27, 2023, and the six-week study block was conducted February 28-April 6, 2023. A total of 25 students provided written consent to participate and responded to the follow-up emails and questionnaires. The 25 students were randomly assigned to the music therapy group or the control group. Six students opted out before further data were collected due to scheduling conflicts. In total, 19 students (7 males, 2 unknown) completed the study: Music Therapy (*n* = 10, 3 males), Control (*n* = 9, 4 males). On average, students in the Music Therapy group attended 4/6 of the weekly sessions.

In block 2 of the study, recruitment was conducted September 8, 2023 - October 15, 2023, and the six-week study block was conducted October 16- November 24, 2023. A total of 143 students provided written consent to participate and responded to the follow-up emails and questionnaires. The 143 students were randomly assigned to the music therapy group, or the control group. The primary researcher’s previous experience of participants in the control group not completing the study informed her decision to allocate more students to the control group than the music therapy group. Four students in the music therapy group opted out before the first session, and two did not complete the study. Three students in the control group opted out during the first week, and five did not complete the study. In total, 129 students completed the study (121 recruited as a course component option); Music Therapy (*n* = 57, male = 9) Control (**n* *= 72, male = 8, prefer not to say = 1). On average, students attended 5/6 of the music therapy sessions over the six-week study period.

Demographically, students from all university Faculties were represented but most students were in the Faculty of Science (80%). 143/148 students self-described their ethnicity, broadly reporting: European/White/Caucasian (*n* = 46), South Asian (*n* = 30), Middle-Eastern (*n* = 13), East-Asian (*n* = 14), Mixed Ethnicity (**n* *= 10), North American (*n* = 6), sub-Saharan African (*n* = 8), Caribbean (*n* = 6), Indigenous (*n* = 3), Black (*n* = 2), Jewish (*n* = 1), Latino (*n* = 2) North African (*n* = 2). Self-descriptions are presented in S1 Table.

### Measures

#### Initial demographic, personality, musical background, and quality of life questionnaires *(completed by Control and Music Therapy Groups).*

The following four background questionnaires were given to all participants in week 1. The Quality of Life questionnaire was repeated in week 6.

1. ***Demographic information*** was collected from each participant through a questionnaire that asked the participants to self-describe gender, year of birth, ethnic origin, use of psychotropic medication, previous or current participation in therapy, and previous or current participation in a music therapy course.
2. ***Ten Item Personality Inventory (TIPI)*** is a standardized self-report questionnaire consisting of ten pairs of words to measure a person’s Big Five personality dimensions: Extraversion, Agreeableness, Conscientiousness, Emotional Stability, and Openness to experiences [35]. Participants rate the extent that each pair of words applies to themselves on a Likert scale from (1) disagree strongly to (7) agree strongly. The TIPI has been shown to have good validity: mean convergent validity with the Big-Five Inventory was r = 0.77 [35].
3. ***Goldsmith Music Sophistication Index (GOLD-MSI)*** is a tool for measuring musical attitudes, behaviors, and skills. It is a self-report questionnaire measuring musical sophistication, defined as musical skills, expertise, achievements, and related behaviors [32]. There are five subscales within the GOLD-MSI: (1) Active Engagement, (2) Perceptual Abilities, (3) Musical Training, (4) Singing Abilities, and (5) Emotional response to music), as well as an overall music sophistication score. A study by Mullensiefen et al. [32] reported that the GOLD-MSI possesses good reliability on each subscale (all α and ω > .79).
4. ***World Health Organization Quality of Life (WHOQOL-BREF)*** is a questionnaire containing 26 questions to assess four domains: (1) Physical Health, (2) Psychological Health, (3) Social Relationships, and (4) Environmental Quality of Life. The WHOQOL-BREF provides a valid and reliable alternative to the assessment of domain profiles using the WHOQOL-100 [36].

#### Measures taken in week 1 and week 6 (completed by Control and Music Therapy Groups).

Three questionnaires and two physiological measures were taken in week 1 and again in week 6.

**Questionnaires**

1. ***Perceived Stress Scale (PSS-10)*** is a 10-item self-report questionnaire designed to evaluate the extent to which an individual perceives life to be “unpredictable uncontrollable and overloading” [37]. The scale is designed to assess feelings about life events and situations over the previous month using a five-point scale ranging from (0) Never to (4) Very Often. PSS scores have demonstrated adequate reliability (α = .78) and moderate concurrent criterion validity with the amount of stress experienced during an average week (*r* = .39 p < .001) and the frequency of stressful life events within the past year (*r* = .32 p < .001) [38]. Additional studies reporting the PSS-10 to have good internal consistency and reliability include Barbosa-Leiker et al. [39] Golden-Kreutz et al. [40] and Reis et al. [41].
2. ***State Trait Anxiety Inventory - State Version (STAI-S)*** includes twenty questions reporting the intensity of participant anxiety at the moment of testing [42]. The STAI-S was administered in the present study to measure how students’ anxiety changes as a result of external factors in the moment. When completing the STAI-S, participants rate the intensity of their feelings on a scale from (1) not at all to (4) very much so. The STAI-S has shown good reliability and validity across the different normative groups; Cronbach’s alpha = 0.86-0.95 [42]. Construct validity was established in two studies by comparing the mean STAI-S scores of college students in anxiety-inducing conditions [42].
3. ***Self-rated stress scale (1–5).*** Participants rated their stress from 1-5 (1 = None 2 = Mild 3 = Moderate 4 = High 5 = Extreme).

**Physiological Measures**

1. ***Cortisol*** is a glucocorticoid secreted from the adrenal glands that is often used as a biomarker for stress [25]. Hair cortisol acts as a proxy for total HPA activity in the preceding months; approximately one centimeter of hair growth from the scalp represents a preceding month of cortisol released [25]. Cortisol from hair samples thus provides information about participant HPA activity retrospectively. Several studies have shown that hair cortisol levels can serve as a reliable approximation of average blood cortisol levels, pointing to the validity of this method relative to established standards [25,43]. In our study, hair samples were collected from participants as per the following steps: (1) cut a small sample of hair and place it on the paper provided in the kit, (2) fold the paper, and place the paper with the hair in the envelope provided (S1 File). Hair samples were sent to the Drug Safety Laboratory, Robarts Research Institute, Western University, Ontario for analysis. The process implemented to extract the cortisol is provided in S2 File.
2. ***Heart rate variability (HRV)*** is a non-invasive measure of the autonomic nervous system as a reliable assessment of stress [26]. Greater variability in heart rate can result in a greater ability to rapidly cope with uncertain and changing environments [27]. In this study, HRV was collected using the Welltory smart phone application using the camera of a Samsung tablet or smart phone (S3 File). HRV data was measured using Standard Deviation of Normal-to-Normal Intervals (SDNN), which is a measure used to assess the variation in time intervals between normal heart beats. Participants placed their finger over the phone camera and flash for two minutes. Correlation analysis has shown an almost perfect correlation of the Welltory phone application against the ECG gold standard [44,45].

#### Measures taken before and after each music therapy session (Music Therapy Group only).

Two of the questionnaires and one of the physiological measures were also taken in the Music Therapy group before and after each music therapy session: STAI-S, Self-rated stress scale (1–5), and HRV.

#### Measures taken after six-weeks of music therapy (Music Therapy Group only).

A feedback survey was completed by participants in the music therapy group consisting of both closed and open questions.

### Procedure

Students were recruited in two blocks from across campus via printed posters and social media platforms. In addition to the required study detail, the recruitment material communicated that participants would have a choice to participate in a draw for a chance to win one of five prizes of $50. Block 1of the study was offered during the winter semester, and block 2 of the study was offered in the Fall semester. An additional form of recruitment was added in block 2, in which students were also recruited from the course HUMBEHAV_2AP3 as an optional component of the course. Potential participants accessed the *Letter of Information* and the *Consent Form* as a Google form via email or a QR code. Students who provided consent received an ID number to complete an online demographic survey, the Goldsmith Music Sophistication Index (GOLD-MSI), the Ten Item Personality Inventory (TIPI) and the World Health Organization Quality of Life BREF (WHO-QOL- BREF). Given these measures were administered online and analyzed by research assistants who were blind to group assignments, both the administration and analyses were blinded. Participants were randomly assigned to the music therapy group or the control group. More participants were allocated to the control group than the music therapy group in anticipation of attrition based on a previous study [8]. Participants in the music therapy group were provided a feedback survey at the end of the study, and participants in the control group were provided the opportunity to participate in one or two music therapy groups at the end of the six-week study period.

#### Music therapy group.

Each group music therapy session was conducted in a private room in the University Student Centre building. The music therapists facilitating the music therapy groups were all credentialed and in good standing with the Canadian Association for Music Therapists. To minimize facilitator effects, five different music therapists facilitated the groups. A total of 21 undergraduate student research assistants, who were either completing a research project course for credit or volunteering, assisted with data collection (S4 File).

Upon arriving at the music therapy room, participants were asked to sit in one of the chairs that had been placed in a circle in the room. Research assistants provided each of the participants with a Samsung Tablet (or a link) which had been pre-loaded with the questionnaires to be filled out. In weeks 1 and 6, this included the PSS, STAI-S, and Self-rated stress scale. HRV was also taken using the Welltory Application on the tablets. The de-identified data from all the questionnaires were automatically input into a Google spreadsheet.

In weeks 1 and 6, the research assistants also collected a hair sample from each participant in the music therapy group as per the following steps: (1) cut a small sample of hair and place it on the paper provided in the kit, (2) fold the paper, and place the paper with the hair in the envelope provided. Hair samples were sent to the Drug Safety Laboratory at Western University, Ontario (S1 File).

In all 6 weeks, the Research Assistants gave the participants the tablets before and after each music therapy session to fill out the STAI-S and Self-rated stress scale as well as to record their HRV.

After the initial data had been collected, the research assistants left the music therapy room, and the music therapist conducted a 45-minute music therapy session. After each group music therapy session, the research assistants returned to the music therapy room to collect the end-of-session data. The interventions implemented in the music therapy group included listening to music, song writing, singing, lyric analysis, playing instruments, improvising, and verbal processing. The music therapy groups were informed by the model offered at the McMaster Student Wellness Centre, *Stress Less* group, and Open Circle’s guidelines (S5 File). More specific examples of interventions used in the music therapy group can be found in S6 File.

In block 1 of the study, one music therapy group was offered each week (~ 10 participants/group) for six-weeks, and in block 2 of the study, six music therapy groups were offered each week (~ 10 participants/group) for six-weeks.

#### Control group.

Participants in the control group were contacted via email in week 1 and week 6 of the study to schedule a day and time to come in to complete the Perceived Stress Scale, the STAI-S, and the self-rated stress scale (all via the Samsung tablets). At this time, their HRV was also recorded, and a hair sample collected for cortisol analysis. As with the Music Therapy Group, the de-identified data were automatically input into a Google spreadsheet.

#### Blinding.

Blinding was put in place where possible within the design of the experiment. All data analyses were conducted by research assistants who were blind to group assignments. The demographic survey, the Goldsmith Music Sophistication Index (GOLD-MSI), the Ten Item Personality Inventory (TIPI) and the World Health Organization Quality of Life BREF (WHO-QOL- BREF) were administered online prior to the onset of the intervention, and thus were administered in a blinded context. In weeks 1 and 6, the Perceived Stress Scale, the STAI-S, the self-rated stress scale, HRV, and hair cortisol were administered in person, so the research assistants who collected this data were aware of which group participants were in (although it should be noted that the questionnaires were administered on tablets, so the research assistants were not privy to how participants filled them out). However, the data analyses were conducted by different research assistants who were blind to group assignments. In the music therapy group only, the STAI-S, self-rated stress scale, and HRV were collected at the beginning and end of each music therapy session. The research assistants administering these were not blind; however, these measures were not collected each week in the control group, and the analyses of changes pre- to post individual therapy sessions were of course only conducted in the music therapy group.

### Analysis plan

Analyses were conducted using JASP 0.14.1 for the linear models, and RStudio 2022.07.02 for the descriptives, correlations, t-tests, and the linear models for the pre-post music therapy scores across the six-weeks of music therapy.

#### Potential co-variables.

Initial exploratory linear regression models examined the relationships between each of the outcome variables, and the demographic data (gender, year of birth, Faculty of Study, current or previous use of medication, in-progress or completion of Introduction to Music Therapy Course, and previous or current engagement in therapy). Additionally, exploratory correlations were examined between the outcome variables and each of the WHOQOL-BREF categories and GOLD-MSI subcategories to determine whether any of these should be included in the main analyses.

Correlations were conducted for each of the week-six WHOQOL- BREF categories (Physical Health, Psychological Health, Social Relationships, and Environmental Health), in relation to changes in the stress and anxiety measures (cortisol, perceived stress, state anxiety, self-rated stress, and heart rate variability). The week-six WHOQOL-BREF responses are reflective of the study period. Bayesian correlations were conducted for each of the five TIPI categories (1. Openness, 2. Conscientiousness, 3. Extraversion, 4. Agreeableness, and 5. Emotional Stability), in relation to the changes in each stress and anxiety measure (cortisol, perceived stress, state anxiety, self-rated stress, and heart rate variability), and for each of the six subscales of the GOLD-MSI categories (1. Active Engagement, 2. Perceptual Abilities, 3. Musical Training, 4. Singing Abilities, 5. Emotional response to music, and 6. general music sophistication), in relation to the changes in each stress and anxiety measures (cortisol, perceived stress, state anxiety, self-rated stress, and heart rate variability). Bayesian correlations were implemented as we hypothesized that neither personality types nor music sophistication would be related to therapy outcomes [46].

#### Two main questions.

Question 1: *Does participating in a 45-minute group music therapy session reduce stress and anxiety from before to after the session?*

In the experimental group, linear mixed effect models were conducted to determine if there was a reduction in stress (self-rated stress scale (1–5), and anxiety (STAI-S) scores from before to after group music therapy sessions, as well as to determine if there was an average increase in heart rate variability from before to after group music therapy sessions, across the six-weeks. The models consisted of ‘participants’ as the random intercept, and the random slopes included ‘Week’ and pre-post music therapy measures.

Question 2: *Does participating in six weeks of weekly group music therapy sessions reduce stress and anxiety in comparison to the control group?*

One-tailed (between groups) Student’s *t-*tests or Mann-Whitney-Wilcoxon test (if change data not normally distributed) were conducted to determine if changes from week 1 to week 6 in STAI-S scores, self-rated stress scale (1–5), heart rate variability, perceived stress scale, and cortisol scores were significantly different between the music therapy and control group.

#### Two secondary questions.

Question 1: Do participants in music therapy report finding the intervention helpful for managing stress and anxiety?

A feedback form was provided to participants in the music therapy groups after the six-week block of music therapy. Descriptives were gathered for yes/no questions, and thematic analyses were conducted for open ended questions implementing a deductive and semantic approach.

Question 2: Would participants engage in music therapy if it was offered on campus?

Participants in the control group were provided an opportunity to participate in 1 or 2 music therapy groups via a google poll invitation. Results of the invitation were recorded, as well as the number attending.

## Results

### Potential co-variables

Linear models revealed a weak influence of demographic variables in relation to the changes in the stress and anxiety outcome scores (S2 Table). Regarding the four WHOQOL-BREF categories, significant correlations (*p* < .05) were found between changes in anxiety scores and week-six Physical Health scores (*r* = 0.19), changes in anxiety and week-six Social Relationships (*r* = 0.24), and changes in stress and week-six Social Relationships (*r* = 0.29). After Bonferroni corrections, only the correlation between changes in stress (1–5) and week-six Social Relationships remained significant (r(122) = 0.29, *p* = .02). The week-six Social Relationships data did not meet the assumptions of normality; therefore, the Mann-Whitney U-test was conducted to compare week-six Social Relationship scores between the music therapy group and the control group. The test revealed no significant difference in Social Relationships between the two groups, W = 2385.5, *p* = .25 (S3 Table). Please see S1 Fig for individual changes in WHOQOL-BREF scores by group. Therefore, none of the demographic variables or WHOQOL-BREF categories were included in the main analyses.

We conducted Bayesian correlations to explore the relationships between the five personality categories (TIPI) and the six GOLD-MSI categories with the outcome variables of change in anxiety, self-rated stress (1–5), PSS, and cortisol, under the hypothesis of no relation. Bayesian correlations provided anecdotal to moderate evidence against all of these correlations (BF_10_ 0.11-0.38), with the exception of changes in the variable state anxiety (STAI-S scores) and the GOLD-MSI category of Emotional Response to Music (BF_10_ 1.8, *r* = -0.215), which only provided anecdotal evidence for a weak negative correlation (S4 and S5 Tables). Therefore, we did not include any of the potential co-variables in the analyses.

#### Two main questions.

**Question 1: Does participating in a 45-minute group music therapy session reduce stress and anxiety from before to after the session?**

STAI-S, self-rated stress scores (1–5), and HRV were collected before and after each music therapy group session (*n* = 67). The HRV data were not analysed beyond reporting descriptives due to questionable results and challenges incurred with the Welltory app (S6 Table). The average HRV using SDNN for a healthy 20 year old is 153 + /- 44 ms [47], and according to the Welltory website 149 ms is the average SDNN for young adults [48]. In the present study the average SDNN of participants in week 1, in the music therapy group was 58 + /- 43 ms, with a range of 11ms - 319ms. This topic is discussed further in the Limitations section.

**Fig 1:**
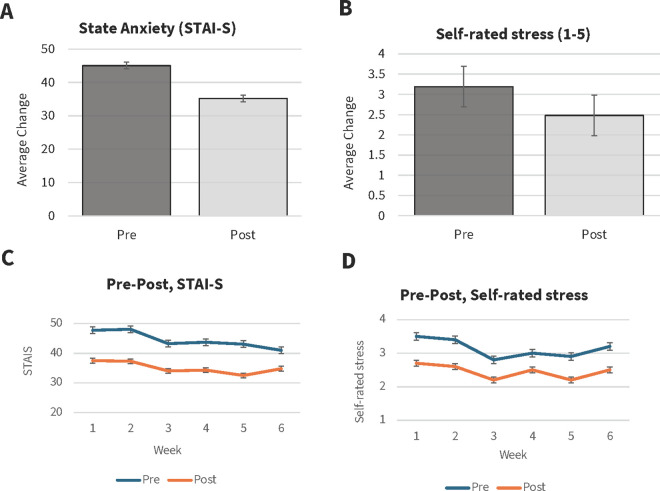
Average Changes in (A) state anxiety (STAI-S) and (B) self-rated stress from pre- to post- each music therapy session and average pre-post scores across weeks in (C) STAI-S and (D) self-rated stress. Error bars reflect ±1 standard error of the mean.

Linear mixed effects models were used to analyse the pre-post STAI-S and self-rated stress (1–5) scores that were collected across weeks (1–6). These models revealed significant reductions from pre-post group music therapy sessions in STAI-S, F(1, 341.7) = 82.03, *p* < .001, and self-rated stress scores (F(1, 377) = 48.8, *p* < .001), with large effect sizes (STAI-S, rank-biserial correlation, 0.91; and self-rated stress, rank-biserial correlation 0.85). A significant effect of Week in STAI-S, F(1, 59.8) = 29.96, **p* *< .001, and self-rated stress scores, F(1, 58) = 13.5, **p* *< .001, was also observed (Fig 1). Additionally, an interaction was observed between Week and STAI-S scores, F(1,445.3) = 4.1, *p* = .04, but not the self-rated stress scores, F(1, 426.9)= 2.1, *p* = .15. The main effects of week indicate that participants’ anxiety and stress fluctuated from week to week. The significant interaction indicates that the magnitude of the benefit of the music therapy sessions for anxiety also varied across weeks. Because these fluctuations were consistent enough across individuals to produce significant effects, they likely reflect varying demands from week to week as the university term progressed. Weekly changes in STAI-S and self-rated stress are reported in S7 Table.

**Question 2: Does participating in six weeks of weekly group music therapy sessions reduce stress and anxiety in comparison to the control group?**

**Fig 2:**
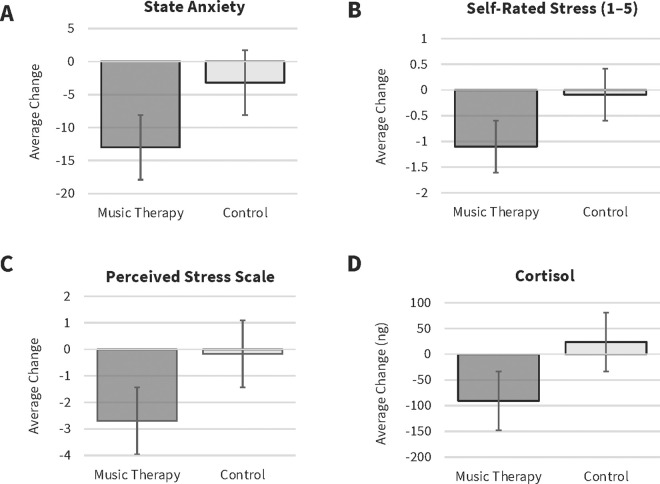
Week 1 to Week 6 changes in STAI-S, Self-rated stress, PSS and Cortisol. Average change in (A) state anxiety (STAI-S), (B) self-rated stress (C) Perceived Stress Scale and (D) cortisol by group, from week 1 to week 6. Error bars reflect ±1 standard error of the mean.

A total of 125 students (music therapy group *n* = 58; control group *n* = 67) completed the STAI-S in both week 1 and week 6. All change scores data met the assumptions of normality as per the Shapiro Wilk test. A one-tailed Student *t*-test comparing the average change in the STAI-S scores from week 1 to week 6 between the music therapy group and the control group revealed a reduction in STAI-S scores in the music therapy group in comparison to the control group, with a large effect size, *t*(123) = 5.6, *p* = < .001, **d* *= 1.02 (Fig 2A).

A total of 125 students (music therapy group (*n* = 58), control group (*n* = 67)) completed the self-rated stress scale in both week 1 and week 6. The change scores did not meet the assumptions of normality as per the Shapiro Wilk test. The Mann-Whitney U test was used to compare changes in self-rated stress scores from week 1 to week 6 between the music therapy group and the control group, revealing a significant reduction in perceived stress in the music therapy group in comparison to the control group, with a medium effect size, *W* = 3094, *p* = < .001, **r* *= 0.59 (Fig 2B).

A total of 135 students (music therapy group (*n* = 63), control group (*n* = 72)) completed the perceived stress scale in both week 1 and week 6. The change scores met the assumptions of normality as per the Shapiro Wilk test. A one-tailed Student *t*-test comparing the average change in the perceived stress scale from week 1 to week 6 between the music therapy group and the control group revealed a reduction in perceived stress in the music therapy group in comparison to the control group with a medium effect size, *t*(133) 2.9, *p* = .002, **d* *= 0.51 (Fig 2C).

A total of 116 students (music therapy group *n = *56; control group *n = *60), provided paired hair samples in both week 1 and week 6. The change scores did not meet the assumptions of normality as per the Shapiro-Wilk test. Therefore, the one-tailed Mann-Whitney U test was used to compare average change scores in cortisol from week 1 to week 6 between the music therapy group and the control group. This revealed a reduction in cortisol in the music therapy group in comparison to the control group, with a small effect size, *W* = 2042, **p* *= .023, *r* = 0.22 (Fig 2D).

It should be noted that the average cortisol levels in the music therapy group were higher at baseline (266.3ng) than the control group (213.7 ng), but due to the non-normality of the data, further exploration of the cortisol change scores using an ANOVA or linear model would not be appropriate. However, further exploration of the baseline scores was conducted using the Mann-Whitney U test, revealing a non-significant difference between groups at baseline, *W* = 1338, **p* *= .06. To further explore average cortisol at baseline for the music therapy and control group a Bayesian Mann-Whitney U test was conducted, revealing anecdotal evidence for no difference between groups at baseline (Bayes Factor 0.98).

Please see S2 Fig for individual changes in each of the four stress and anxiety measures from week 1 to week 6, by group. One outlier in the measure of cortisol can be observed (S2D Fig), however, the difference between groups remains significant with its removal. Individual data points for the stress and anxiety measures can be found on the Open Science Framework https://shorturl.at/ALmT7 and https://shorturl.at/YUmjh.

#### Two secondary questions.

**Question 1: Do participants in music therapy report finding the intervention helpful for managing stress and anxiety?**

**Fig 3:**
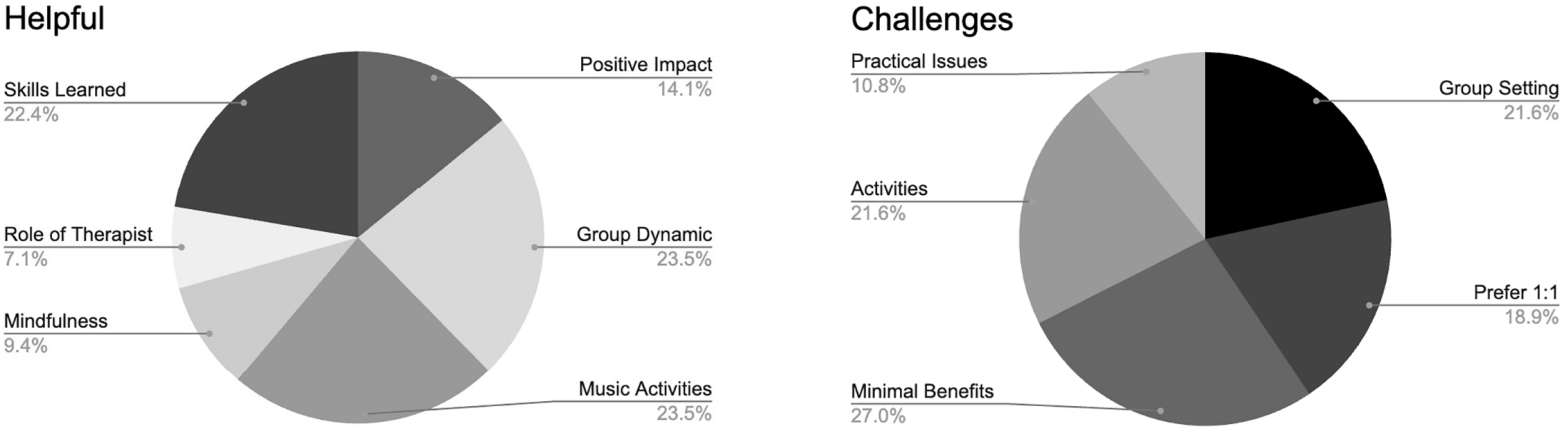
Six themes related to group music therapy being helpful (total comments = 85); and five themes related to the challenges of engaging in group music therapy (total comments = 37).

Music therapy participants (*n* = 68) completed a feedback form, which consisted of five questions. 71% of students responded that they found group music therapy helpful, and 66% responded that they would continue to participate in group music therapy if offered on campus. 122 comments were identified in response to an open-ended question (Question 2) which asked students what they found helpful/unhelpful. A thematic analysis was conducted on these responses revealing six subthemes related to helpfulness and five subthemes related to challenges (Fig 3). Responses to the five questions in the feedback form are displayed in S8 Table. Please refer to S7 File for individual responses.

**Question 2: Would participants engage in music therapy if it was offered on campus?**

Participants in the control group in Block 1 were provided the option to be in the music therapy group in Block 2 which was scheduled to take place in May 2023. Unfortunately, as there are fewer students on campus in the Spring semester, we held Block 2 in the Fall semester (October 2023). Block 2 was the final Block of the study and participants in the control group of Block 2 were offered the option to participate in one or two group music therapy sessions after the conclusion of the study. A total of 49/72 (68%) of participants from the control group in Block 2 responded to an email that included a google form asking their interest and availability to participate in group music therapy sessions. Five different dates and times were offered (the same days of the week and times that the group music therapy sessions had been offered during the six -week study period). 6/49 participants indicated they did not want to participate in a music therapy group. 32 participants indicated that they would like to attend one session, but 6/32 were unable to attend the dates offered. 11 indicated they would like to attend two sessions, but 4/11 were not available on the dates offered. In the end, 22/26 (85%) of the students who indicated they were interested and available to attend one session, actually attended one group music therapy session, and 4/7 (57%) of the students who indicated they were interested and available to attend two sessions, actually attended two group music therapy sessions.

### Post-hoc question

Data about quality of life were collected to determine if any of the quality-of-life categories needed to be considered as covariates in the stress and anxiety analyses. Week-6 data from the WHOQOL-BREF did not correlate with any of the stress and anxiety outcomes, but an average increase in quality-of-life scores was observed in the music therapy group and an average decrease in quality-of-life scores was observed in the control group over the 6-week period of the study. An exploratory post-hoc question was therefore developed: *Is there a difference in change scores across the 6 weeks of the intervention in any of the WHOQOL-BREF categories between the music therapy and control group?* If yes, these observations could suggest that engaging in music therapy positively influences ratings of quality of life.

A total of 131 students (music therapy group (*n* = 62), control group (*n* = 69)) completed the WHOQOL-BREF in both week 1 and week 6. The WHOQOL-BREF data are reported in four different categories: (1) Physical Health, (2) Psychological Health, (3) Social Relationships and (4) Environmental Health. Independent t-tests were conducted between the music therapy and control group comparing change scores of each of the four categories of quality of life to determine if any of these increases were significant. This revealed a significantly greater increase in participants’ Psychological Health scores in the music therapy group in comparison to the control group, with a medium effect size (*t*(130)=-2.57, *p* = .01, *d* = 0.45) (S9 Table). Please see S1 Fig for individual changes in WHOQOL-BREF categories by group.

## Discussion

The offerings of mental health interventions for undergraduate students on university campus are often reactive rather than proactive. Reactive, meaning that the interventions are designed for a specific diagnosis, or students in crisis; and proactive, meaning that the interventions are designed for students who do not have a diagnosis and are not experiencing a crisis, but want to engage in mental health supports to manage their mental wellness. In recognition of the paucity of research exploring proactive interventions for wellness on campus, the present randomized controlled trial was conducted to explore the use of group music therapy to proactively manage undergraduate university students’ stress and anxiety. Considering that the highest levels of anxiety (33.5%) and depression (27.7%) are observed among younger Canadians (15–39 years of age) in comparison to other age groups [49], and that globally suicide is the fourth leading cause of death among youth aged 15–29 [50], preventative and proactive strategies for undergraduate university students are required.

In the present study, we found that participating in a 45-minute group music therapy session reduced stress and anxiety, as measured by changes in the STAI-S and self-rated stress of participants from before to after individual sessions. These observations provide support for immediate, short-term relief of stress and anxiety. This is important as experiencing low levels of stress, even for a short period of time, can have benefits [28]. Further, over the six weeks of the study, stress and anxiety decreased to a greater extent in those who participated in the six weeks of therapy in comparison to those in a student-life-as-usual control group, as measured by the STAI-S, self-rated stress, PSS, and hair cortisol. Thus, both self-report and physiological measures indicated that group music therapy is effective in a proactive group setting.

Despite random assignment to group, hair cortisol was higher on average at the beginning of the study in the participants in the music therapy group compared to those in the control group, although these baseline measures were not statistically significant between groups. Analysing cortisol from hair samples is still a relatively new measure [51], and there are no established normative scores for cortisol. As such, we analysed the average within-participant changes in cortisol levels between groups, as opposed to comparing the average cortisol levels between groups at the end of the study. This within-participant study design also helped to reduce potential variability associated with demographic factors, hair color, and hair washing regimes [52].

The present study also asked participants in the music therapy group about their experience of music therapy, and if they would continue to seek out music therapy if it was offered on campus. 71% of the participants in the music therapy group reported that they found music therapy helpful for proactive management of stress and anxiety, and 66% stated that they would continue to seek out music therapy if it was offered on campus. Participants in the control group were offered the option to participate in group music therapy after the completion of the study period. 68% of the control participants expressed interest, and 79% actually attended a music therapy group (a number had scheduling conflicts), providing confirmation of interest in group music therapy. Together, these responses highlight that students are interested in options beyond verbal-therapies, and the importance of offering more choices for student wellness on campus. Providing more choices to engage in wellness enhances student autonomy and offering supports inclusive of music can help to reduce the stigma associated with therapy, as music is often associated with healthy and socially acceptable activities [7].

The present research study also highlights the potential universal benefits of group music therapy despite participant differences in demographics, music sophistication scores, and personality traits. Using inferential and Bayesian statistics, we found that changes in stress and anxiety over the six-week study period did not significantly correlate with any of the demographic information collected, including gender, ethnicity, area of study, age, or previous/current engagement in therapy/medications. Regarding differences in music sophistication scores, the profession of music therapy is based on an assumption that all humans can engage in music therapy regardless of background or experience with music, despite music being the therapeutic tool through the use of singing, song writing, listening, improvising, and lyric analysis. After conducting Bayesian correlations between the six GOLD-MSI categories and changes in the stress and anxiety measures, the low Bayes Factors indicated anecdotal to moderate evidence that changes in stress and anxiety scores did not differ as a result of music sophistication (GOLD-MSI scores). To our knowledge, this is the first study to explore correlations between the GOLD-MSI domains and changes in stress and anxiety as a result of participation in a therapy context. The third potential covariate that we explored was personality traits. Previous research suggests that personality domains are correlated with an individual’s propensity for stress management [29]. In our study, Bayesian correlations indicated anecdotal to moderate evidence for weak correlations between the different personality traits and changes in stress and anxiety measures. There is a paucity of research about personality traits and changes in stress or anxiety pre-post therapy. Our research contributes to this gap and supports that personality domains are not strong predictors of therapy outcomes [30,31]. Collectively, the exploration of these three potential covariates demonstrates that individual differences in demographics, music sophistication, and personality traits, are not strong predictors of successful outcomes in group music therapy, highlighting its universal effect.

In addition to its effects, accessibility, and universality, group music therapy is cost-effective. By accommodating up to 10 students per session, group music therapy not only addresses individual needs, but also alleviates strains on overall campus support systems. This proactive approach helps to mitigate crisis events and foster a campus culture of wellness, thereby promoting a financially viable healthcare model [53,54]. The role of stress in adverse mental and physical health outcomes underscores the importance of proactive group interventions. Research has shown that proactive stress management can reduce the risk of conditions such as dementia [55], diabetes [56], and coronary heart disease [57]. As well, it can assist with symptom management, potentially minimizing the need for costly supports associated with conditions like dementia [58].

To support proactive wellness options on campus to manage stress and anxiety, universities might consider including mental health options and experiences as part of course curricula. This can help to normalize proactive mental health practices and remove logistical barriers to accessing support. For instance, in our study, many students were recruited through a course offering that included an experiential component related to mental health. By incorporating wellness activities into course requirements, students can access mental health support without the burden of scheduling additional time amidst their academic and extracurricular commitments. The promotion of mental health opportunities by professors is essential for fostering a proactive wellness culture on campus. By making mental health supports logistically accessible and integrating them into academic programs, universities can play a vital role in promoting student well-being.

Overall, integrating stress management strategies like group music therapy on university campuses is effective for stress and anxiety management, is universally accessible, promotes a positive mental health campus culture, and offers a cost-effective solution to the increasing challenge of meeting the mental health needs of students.

### Limitations

There were a few reasons why not all data were collected from all participants. First, regarding the collection of the hair samples, some participants did not feel comfortable having a research assistant cut a sample of hair. And some who did provide the first sample, did not feel comfortable providing a second hair sample six weeks later. While this reduced the total number of paired hair samples we could analyze, the sample size was still large enough to meet the a priori power analysis. Second, and more seriously, the heart rate variability data was sufficiently noisy that it was unanalyzable. As we needed a simple, cost-effective, portable option, we chose the free version of the Welltory Application [44,45]. However, as described above, the data were not reliable, perhaps because the readings took place within a group setting, where there was talking and distractors, despite attempts to keep this to a minimum. Collecting HRV across participants with different devices could have also contributed to the variability. For future applications, it would likely be better to use individual application licenses or a more traditional measure of heart rate. Third, data were not collected if a participant arrived late to a music therapy session, or needed to leave early, as the group nature of the sessions did not allow time flexibility. Therefore, the participation numbers for some weeks are higher than the data points collected. As well, although attendance to the music therapy groups was generally good, there was a bus strike and several student protests during the study period, which likely effected attendance. Fourth, females are overrepresented in this study, which is reflective of undergraduate students at the university. Finally, recognizing that the control group was engaging in ‘student life as usual’, it is possible that the results of this study are reflective of the Hawthorne effect, that is, simply being in any intervention might have produced an effect. However, previous research has found music therapy to be as effective, or to outperform the standard of care [8–11]. Our research question was specific to exploring group music therapy as an option for students to proactively manage their stress and anxiety, opposed to comparing its effects against a different intervention or activity. It remains for future research to determine the critical elements of group music therapy interventions (e.g., use of music; combination of music and verbal elements; use of musical instruments; a skilled psychotherapist rather than a music teacher) for students to effectively manage their stress and anxiety.

## Conclusion

The present randomized control trial highlights the benefits of offering group music therapy on campus to undergraduate university students as a proactive intervention for stress and anxiety related to student life. Significant average reductions in all measures of stress and anxiety were observed from week 1 to week 6 in the music therapy group in comparison to the control group. Specifically, this included the self-report questionnaires STAI-S, self-rated stress (1–5), and Perceived Stress Scale, as well as the physiological hair cortisol measure. Significant average reductions in anxiety (STAI-S) and self-rated stress (1–5) were also observed from before to after each of the group music therapy sessions. Outcomes were largely independent of demographic variables, music sophistication, or personality traits, suggesting that group music therapy can positively affect stress and anxiety outcomes regardless of differences in these domains. This study elucidates the effectiveness of group music therapy and the benefits of including group music therapy as part of a proactive student wellness campus culture.

## Supporting information

S1 Fig WHOQOL-BREF scores by group. Individual changes in (A) Physical Health, (B) Psychological Health, (C) Social Relationships and (D) Environmental Health.
(TIFF)

S2 Fig Changes in Stress and Anxiety by group. Individual changes in (A) STAI-S, (B) Self-rated Stress, (C) PSS and (D) Cortisol by group.
(TIFF)

S1 Table Participants’ Ethnicity. Each participant self-described their ethnicity, no edits made by authors.
(PDF)

S2 Table Linear models (Stress/anxiety and demographic data). Linear model summaries including demographic variables (gender, year of birth, Faculty of Study, current or past use of psychotropic medication, currently attending or completed Introduction to Music Therapy course, past or present engagement in therapy) in relation to each stress and anxiety outcome (A. STAI-S, B. Stress 1–5, C. Perceived Stress Scale, D. Cortisol).
(PDF)

S3 Table Pearson correlations (Stress/anxiety and WHO-QOL). Pearson correlations between changes in stress/anxiety scores and week-6 WHO-QOL scores.
(PDF)

S4 Table Bayesian Pearson correlations (Stress/anxiety and TIPI). Bayesian Pearson correlations between changes in stress/anxiety scores and personality categories (TIPI).
(PDF)

S5 Table Bayesian correlations (Stress/anxiety and GOLD-MSI). Bayesian Pearson Correlations between changes in stress/anxiety scores and music sophistication (GOLD).
(PDF)

S6 Table Heart Rate Variability. Descriptive Statistics of heart rate variability before and after each group music therapy session.
(PDF)

S7 Table Pre-post music therapy (Stress/Anxiety). Changes in state anxiety and self-rated stress from pre-post each group music therapy session.
(PDF)

S8 Table Music therapy participants’ feedback. Participants’ responses to the feedback questionnaire after six-weeks of music therapy.
(PDF)

S9 Table World Health Organization Quality of Life BREF Scores. Participants’ average scores from week 1 to week 6 in each of the four WHO-QOL categories.
(PDF)

S1 File Hair Collection Protocol. (PDF)

S2 File Extraction of cortisol from hair samples, The Drug Safety Lab. (PDF)

S3 File Types of smart phones utilized by Welltory application for HRV data. (PDF)

S4 File Student Research Assistants and roles. (PDF)

S5 File McMaster Wellness Centre *Stress Less* Group & Open Circle Guidelines. (PDF)

S6 File Examples of specific Music Therapy interventions. (PDF)

S7 File Music therapy participants’ individual responses to Question 2 of feedback form. (PDF)
