# Supplementary figures and images for: Group music therapy for the proactive management of stress and anxiety
Source: PLOS Ment Health. 2025 Aug 14;2(8):e0000312. doi: 10.1371/journal.pmen.0000312 (PMC12798455; doi:10.1371/journal.pmen.0000312)

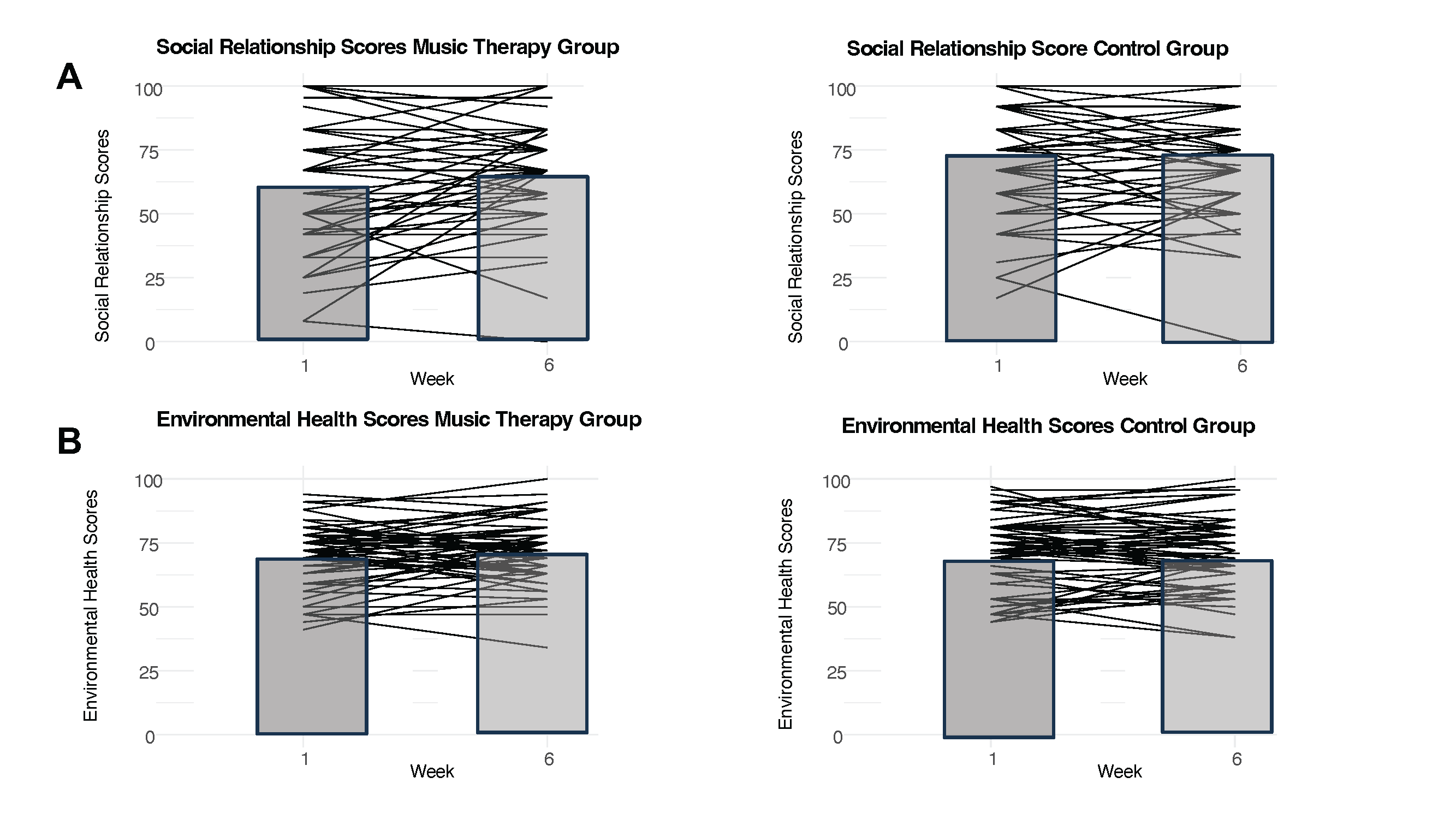

Supplement: S1 Fig — Individual changes in (A) Physical Health, (B) Psychological Health, (C) Social Relationships and (D) Environmental Health. (TIFF) [file pmen.0000312.s001.tiff]

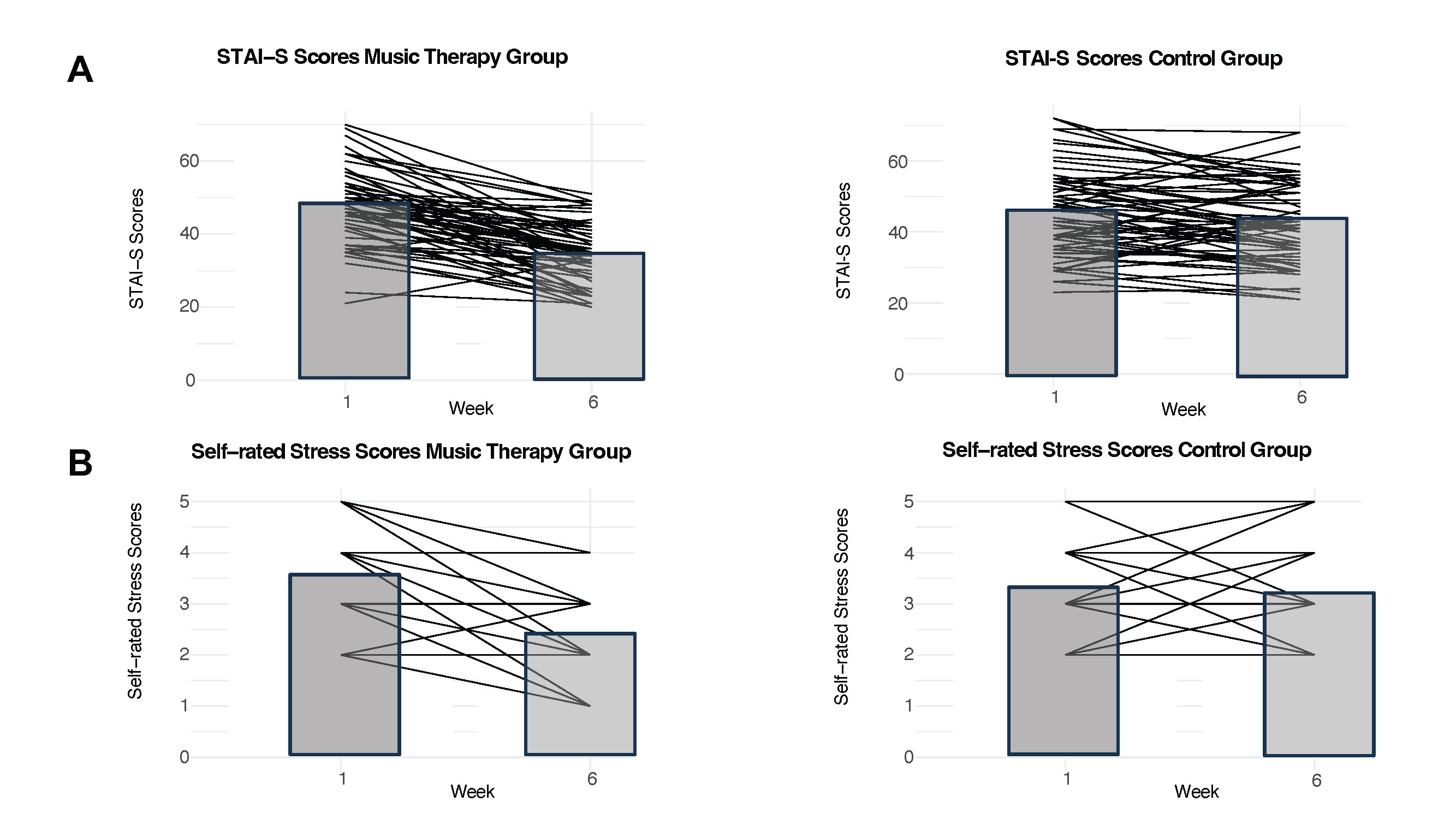

Supplement: S2 Fig — Individual changes in (A) STAI-S, (B) Self-rated Stress, (C) PSS and (D) Cortisol by group. (TIFF) [file pmen.0000312.s002.tiff]

**S2 File.** Process to extract cortisol from hair sample at The Drug Safety Lab (Greff et al. 2019)

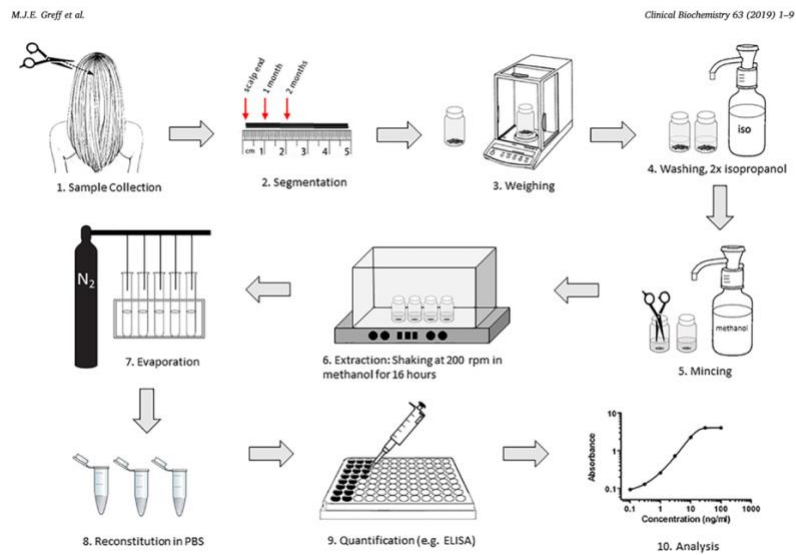

Supplement: S2 File — (PDF) [file pmen.0000312.s013.pdf]
