# Supplementary material for: Group music therapy for the proactive management of stress and anxiety
Source: PLOS Ment Health. 2025 Aug 14;2(8):e0000312. doi: 10.1371/journal.pmen.0000312 (PMC12798455; doi:10.1371/journal.pmen.0000312)
Supplement: S1 Table — Each participant self-described their ethnicity, no edits made by authors. (PDF) [file pmen.0000312.s003.pdf]

**S1 Table.** Participants' Self-Described Ethnicity

| Group | Self-described | Category |
|---|---|---|
| MT | Black | Black |
| C | Black | Black |
| MT | Guyanese | Caribbean |
| MT | Caribbean | Caribbean |
| C | Canadian born – Trinidadian and Congolese | Caribbean |
| C | Jamaica | Caribbean |
| C | ½ Trinidadian ½ Guyanese | Caribbean |
| C | Trinidadian, Jamaican, and Vincentian | Caribbean |
| MT | Chinese | East Asian |
| MT | Chinese | East Asian |
| MT | Chinese | East Asian |
| MT | Filipino | East Asian |
| MT | Korean | East Asian |
| C | Filipino | East Asian |
| C | Chinese | East Asian |
| C | Korean | East Asian |
| C | East Asian | East Asian |
| C | Asian (Chinese) | East Asian |
| C | Chinese | East Asian |
| C | Mainland Chinese, Shan Xi | East Asian |
| C | Korean | East Asian |
| C | East Asian | East Asian |
| MT | European (England and Ireland) | European (white) |
| MT | Italy | European (white) |
| MT | Caucasian (white) | European (white) |
| MT | Caucasian | European (white) |
| MT | White | European (white) |
| MT | White Canadian | European (white) |
| MT | Italian | European (white) |
| MT | Caucasian | European (white) |
| MT | Caucasian | European (white) |
| MT | Caucasian | European (white) |
| MT | Greek and Cypriot Canadian | European (white) |
| MT | Canadian citizen (Greek and Italian ethnicities) | European (white) |
| MT | Romanian | European (white) |

| | | |
|---|---|---|
| MT | White | European (white) |
| MT | British | European (white) |
| MT | Caucasian | European (white) |
| MT | Caucasian | European (white) |
| MT | Caucasian | European (white) |
| MT | Caucasian | European (white) |
| MT | Taiwanese | East Asian |
| MT | White | European (white) |
| MT | European | European (white) |
| MT | European | European (white) |
| MT | White | European (white) |
| MT | White | European (white) |
| MT | White | European (white) |
| C | Caucasian | European (white) |
| C | Spanish/Irish | European (white) |
| C | My background is Romanian, my entire family is from there, but I was born and raised in Canada | European (white) |
| C | European | European (white) |
| C | European | European (white) |
| C | Ukrainian (Caucasian) | European (white) |
| C | White | European (white) |
| C | White | European (white) |
| C | Caucasian | European (white) |
| C | Canadian | European (white) |
| C | European | European (white) |
| C | White | European (white) |
| C | White | European (white) |
| C | Caucasian | European (white) |
| C | European descent | European (white) |
| C | Caucasian | European (white) |
| C | Italian & French Canadian | European (white) |
| C | Caucasian | European (white) |
| C | White (English) | European (white) |
| C | Caucasian, born in Ukraine | European (white) |
| MT | Kichwa-Otavalo Ecuador (indigenous) | Indigenous |
| C | Canadian & Métis | Indigenous |
| C | Indigenous-White | Indigenous |
| C | Jewish | Jewish |
| C | Latina | Latino |

| | | |
|---|---|---|
| C | Latin | Latino |
| MT | Middle eastern | Middle Eastern |
| MT | Lebanese | Middle Eastern |
| MT | I am Syriac Middle Eastern | Middle Eastern |
| MT | Egyptian | Middle Eastern |
| MT | Persian | Middle Eastern |
| MT | Iraqi Turkmen | Middle Eastern |
| MT | Caucasian | European (white) |
| MT | Asian | Middle Eastern |
| C | Born in Saudi, lived in India, now a Canadian citizen | Middle Eastern |
| C | Iranian/Persian | Middle Eastern |
| C | Persian | Middle Eastern |
| C | Iranian | Middle Eastern |
| C | Middle eastern | Middle Eastern |
| MT | Vietnamese Chinese | Mixed Ethnicity |
| MT | French, English and Metis | Mixed Ethnicity |
| C | Italian/ Arab | Mixed Ethnicity |
| C | Indigenous and European Canadian | Mixed Ethnicity |
| C | I am half Japanese, Italian and Irish | Mixed Ethnicity |
| C | Canadian born, parents are Romanian and Armenian. | Mixed Ethnicity |
| C | Caribbean, west Indian | Mixed Ethnicity |
| C | Chinese, Vietnamese | Mixed Ethnicity |
| C | Canadian, Portuguese | Mixed Ethnicity |
| C | Half Filipino with a little bit of Spaniard, Half white: French, English, Scottish, Irish, with a little bit of Ojibwe | Mixed Ethnicity |
| C | Egyptian | North African |
| C | Arab (North African) | North African |
| MT | Canadian | North American |
| MT | Canadian | North American |
| MT | French Canadian | North American |
| C | Canadian | North American |
| C | Canadian | North American |
| C | Canadian | North American |
| MT | Sri-Lankan Canadian | South Asian |
| MT | South Asian (Punjabi) | South Asian |
| MT | South Asian | South Asian |
| MT | Pakistani | South Asian |

| | | |
|---|---|---|
| MT | Bangladeshi | South Asian |
| MT | Pakistani | South Asian |
| MT | Bangladesh, South Asian | South Asian |
| MT | South Asian- Indian | South Asian |
| MT | Indian – Punjabi | South Asian |
| MT | Indian/South Asian | South Asian |
| MT | Sri Lankan | South Asian |
| MT | Asian | South Asian |
| C | Sri Lankan | South Asian |
| C | South Asian | South Asian |
| C | Indian | South Asian |
| C | South Asian | South Asian |
| C | Indian | South Asian |
| C | Pakistani | South Asian |
| C | Filipino | South Asian |
| C | India | South Asian |
| C | South Asian (Pakistani) | South Asian |
| C | Pakistani, south Asian | South Asian |
| C | South Asian | South Asian |
| C | Pakistani – brown | South Asian |
| C | Pakistan | South Asian |
| C | Indian (Punjabi) | South Asian |
| C | Indian (South Asian) | South Asian |
| C | South Indian | South Asian |
| C | Central South Indian | South Asian |
| C | South Asian | South Asian |
| MT | Nigerian | Sub-Saharan African |
| MT | Black Nigerian | Sub-Saharan African |
| MT | African | Sub-Saharan African |
| C | East African – Rwanda, Burundi, Congo (DRC) | Sub-Saharan African |
| C | Congo, DR | Sub-Saharan African |
| C | Nigerian | Sub-Saharan African |
| C | Nigerian | Sub-Saharan African |
| C | Somali | Sub-Saharan African |

*Self-described data were initially independently collated by two student research assistants (S4 File). Data were then collaboratively assigned to a category. MT = Music Therapy Group; C = Control Group.*
