# Supplementary material for: Group music therapy for the proactive management of stress and anxiety
Source: PLOS Ment Health. 2025 Aug 14;2(8):e0000312. doi: 10.1371/journal.pmen.0000312 (PMC12798455; doi:10.1371/journal.pmen.0000312)
Supplement: S2 Table — Linear model summaries including demographic variables (gender, year of birth, Faculty of Study, current or past use of psychotropic medication, currently attending or completed Introduction to Music Therapy course, past or present engagement in therapy) in relation to each stress and anxiety outcome (A. STAI-S, B. Stress 1–5, C. Perceived Stress Scale, D. Cortisol). (PDF) [file pmen.0000312.s004.pdf]

S2 Table. Linear model summaries including demographic variables (gender, year of birth, Faculty of Study, current or past use of psychotropic medication, currently attending or completed Introduction to Music Therapy course, past or present engagement in therapy) in relation to each stress and anxiety outcome (A) STAI-S, (B) Stress 1-5,(C) Perceived Stress Scale, (D) Cortisol.

A.

Model Summary – STAI-S

Model	R	R^2	Adjusted R^2	RMSE
M_0	0.000	0.000	0.000	10.771
M_1	0.350	0.123	-0.011	10.830

Note. M_1 includes Gender, Year of Birth, Faculty, PsychometricMedications(past/present), IntroMusicTherapyCourse (past/present), Therapy(past/present)

B.

Model Summary – Stress (1-5)

Model	R	R^2	Adjusted R^2	RMSE
M_0	0.000	0.000	0.000	1.004
M_1	0.399	0.159	0.031	0.988

Note. M_1 includes Gender, Year of Birth, Faculty, PsychometricMedications(past/present), IntroMusicTherapyCourse (past/present), Therapy(past/present)

C.

Model Summary – Perceived Stress Scale

Model	R	R^2	Adjusted R^2	RMSE
M_0	0.000	0.000	0.000	5.039
M_1	0.280	0.078	-0.042	5.145

Note. M_1 includes Gender, Year of Birth, Faculty, PsychometricMedications(past/present), IntroMusicTherapyCourse (past/present), Therapy(past/present)

D.

Model Summary - Cortisol

Model	R	R^2	Adjusted R^2	RMSE
M_0	0.000	0.000	0.000	412.517
M_1	0.362	0.131	-0.022	416.950

Note. M_1 includes Gender, Year of Birth, Faculty, PsychometricMedications(past/present), IntroMusicTherapyCourse (past/present), Therapy(past/present)
