# Supplementary material for: Group music therapy for the proactive management of stress and anxiety
Source: PLOS Ment Health. 2025 Aug 14;2(8):e0000312. doi: 10.1371/journal.pmen.0000312 (PMC12798455; doi:10.1371/journal.pmen.0000312)
Supplement: S3 Table — Pearson correlations between changes in stress/anxiety scores and week-6 WHO-QOL scores. (PDF) [file pmen.0000312.s005.pdf]

S3 Table. Pearson correlations between changes in stress/anxiety scores and week-6 WHO-QOL scores.

Change Measure	Week 6 WHOQOL	*n*	Pearon's *r*	*p*	Bonferonni
STAI-S	Physical Health	122	0.190	.04	.57
STAI-S	Psychological Health	123	-0.115	.21	1.0
STAI-S	Social Relationships	122	0.241	.01	.11
STAI-S	Environmental Health	122	0.07	.43	1.0
Self-rated stress	Physical Health	122	.11	.23	1.0
Self-rated stress	Psychological Health	123	-0.04	.66	1.0
Self-rated stress	Social Relationships	122	0.29	.001	.02*
Self-rated stress	Environmental Health	122	0.03	.7	1.0
Perceived Stress Scale	Physical Health	129	-0.01	.9	1.0
Perceived Stress Scale	Psychological Health	130	0.02	.84	1.0
Perceived Stress Scale	Social Relationships	129	-0.1	.26	1.0
Perceived Stress Scale	Environmental Health	129	-0.17	0.06	.99
Cortisol	Physical Health	109	-0.15	0.13	1.0
Cortisol	Psychological Health	110	-0.12	.22	1.0
Cortisol	Social Relationships	109	.02	.83	1.0
Cortisol	Environmental Health	109	-.15	.12	1.0
