# Supplementary material for: Group music therapy for the proactive management of stress and anxiety
Source: PLOS Ment Health. 2025 Aug 14;2(8):e0000312. doi: 10.1371/journal.pmen.0000312 (PMC12798455; doi:10.1371/journal.pmen.0000312)
Supplement: S4 Table — Bayesian Pearson correlations between changes in stress/anxiety scores and personality categories (TIPI). (PDF) [file pmen.0000312.s006.pdf]

S4 Table. Bayesian Correlations between changes in stress/anxiety scores and personality categories (TIPI)

Change Measure	TIPI	Sample Size (n)	Pearson's r	Bayes Factor (BF_{10})
STAI-S	Extroversion	99	0.055	0.145
STAI-S	Agreeableness	100	-0.111	0.226
STAI-S	Conscientiousness	100	-0.024	0.129
STAI-S	Emotional Stability	100	0.105	0.214
STAI-S	Openness	100	-0.003	0.125
Self-rate stress (1-5)	Extroversion	99	0.127	0.271
Self-rate stress (1-5)	Agreeableness	100	-0.139	0.317
Self-rate stress (1-5)	Conscientiousness	100	0.043	0.137
Self-rate stress (1-5)	Emotional Stability	100	0.046	0.138
Self-rate stress (1-5)	Openness	100	0.016	0.126
Perceived Stress Scale	Extroversion	106	-0.010	0.122
Perceived Stress Scale	Agreeableness	107	0.074	0.160
Perceived Stress Scale	Conscientiousness	107	-0.027	0.126
Perceived Stress Scale	Emotional Stability	107	0.145	0.363
Perceived Stress Scale	Openness	107	-3.535e -4	0.121
Cortisol	Extroversion	92	-0.021	0.133
Cortisol	Agreeableness	93	-0.097	0.198
Cortisol	Conscientiousness	93	-0.072	0.164
Cortisol	Emotional Stability	93	0.044	0.141
Cortisol	Openness	93	0.104	0.210
