# Supplementary material for: Group music therapy for the proactive management of stress and anxiety
Source: PLOS Ment Health. 2025 Aug 14;2(8):e0000312. doi: 10.1371/journal.pmen.0000312 (PMC12798455; doi:10.1371/journal.pmen.0000312)
Supplement: S5 Table — Bayesian Pearson Correlations between changes in stress/anxiety scores and music sophistication (GOLD). (PDF) [file pmen.0000312.s007.pdf]

S5 Table. Bayesian Correlations between changes in stress/anxiety scores and music sophistication (GOLD-MSI)

Change Score	GOLD MSI Category	Sample Size	Pearson's r	Bayes Factor (BF_{10})
STAI-S	Active Engagement	119	-0.142	0.368
STAI-S	Perceptual Abilities	119	-0.074	0.157
STAI-S	Musical Training	119	0.022	0.118
STAI-S	Emotional Response to Music	119	-0.215	1.763*
STAI-S	Singing Abilities	119	0.043	0.128
STAI-S	General Sophistication	119	-0.056	0.137
Self-rated stress (1-5)	Active Engagement	119	-0.009	0.115
Self-rated stress (1-5)	Perceptual Abilities	119	-0.085	0.173
Self-rated stress (1-5)	Musical Training	119	0.128	0.298
Self-rated stress (1-5)	Emotional Response to Music	119	-0.042	0.127
Self-rated stress (1-5)	Singing Abilities	119	0.043	0.127
Self-rated stress (1-5)	General Sophistication	119	0.014	0.116
Perceived Stress Scale	Active Engagement	126	-0.025	0.116
Perceived Stress Scale	Perceptual Abilities	126	-0.139	0.368
Perceived Stress Scale	Musical Training	126	0.002	0.111
Perceived Stress Scale	Emotional Response to Music	126	-0.058	0.137
Perceived Stress Scale	Singing Abilities	126	-0.034	0.120
Perceived Stress Scale	General Sophistication	126	-0.048	0.128
Cortisol	Active Engagement	111	-0.113	0.237
Cortisol	Perceptual Abilities	111	-0.066	0.150
Cortisol	Musical Training	111	0.146	0.377
Cortisol	Emotional Response to Music	111	-0.032	0.125
Cortisol	Singing Abilities	111	-0.047	0.134
Cortisol	General Sophistication	111	0.021	0.122
