# Supplementary material for: Group music therapy for the proactive management of stress and anxiety
Source: PLOS Ment Health. 2025 Aug 14;2(8):e0000312. doi: 10.1371/journal.pmen.0000312 (PMC12798455; doi:10.1371/journal.pmen.0000312)
Supplement: S6 Table — Descriptive Statistics of heart rate variability before and after each group music therapy session. (PDF) [file pmen.0000312.s008.pdf]

S6 Table. Descriptive Statistics of heart rate variability before and after each group music therapy session

**Week***	***n***	**Average Pre (*SD*)** Heart Rate Variability (SDNN)	**Average Post (*SD*)** Heart Rate Variability (SDNN)
2	58	61.8 (55.55)	61.4 (32.16)
3	54	58.3 (29.92)	59.0 (28.82)
4	53	53.6 (24.50)	67.4 (38.10)
5	47	55.0 (34.91)	58.8 (33.74)
6	59	76.7 (71.35)	71.4 (09.29)

*In week 1, HRV was only collected before the music therapy session, therefore it is not included in this table.
