# Supplementary material for: Group music therapy for the proactive management of stress and anxiety
Source: PLOS Ment Health. 2025 Aug 14;2(8):e0000312. doi: 10.1371/journal.pmen.0000312 (PMC12798455; doi:10.1371/journal.pmen.0000312)
Supplement: S7 Table — Changes in state anxiety and self-rated stress from pre-post each group music therapy session. (PDF) [file pmen.0000312.s009.pdf]

S7 Table. Average STAI-S and self-rated stress scores from pre-post each group music therapy session.

	n	**Pre-Music Therapy** STAI-S mean(SD)	**Post-Music Therapy** STAI-S mean (SD)	*n*	**Pre-Music Therapy** Self-rated stress mean(SD)	**Post-Music Therapy** Self-rated stress mean (SD)
Week 1	63	47.4 (10.7)	37.2(8.7)	62	3.5 (0.8)	2.7 (0.9)
Week 2	58	48.1 (10.8)	37.3 (8.3)	58	3.4 (0.9)	2.6 (0.9)
Week 3	48	43.3 (12.9)	34 (8.1)	47	2.8 (1.0)	2.2 (0.9)
Week 4	48	43.6 (10.9)	34.1 (8.7)	47	3.0 (0.9)	2.5 (1.1)
Week 5	44	43.1(12.3)	32.5 (8.3)	44	2.9 (1.0)	2.2 (0.8)
Week 6	46	41 (10)	34.8 (8.7)	46	3.2 (0.7)	2.6 (0.8)
