# Supplementary material for: Group music therapy for the proactive management of stress and anxiety
Source: PLOS Ment Health. 2025 Aug 14;2(8):e0000312. doi: 10.1371/journal.pmen.0000312 (PMC12798455; doi:10.1371/journal.pmen.0000312)
Supplement: S8 Table — Participants’ responses to the feedback questionnaire after six-weeks of music therapy. (PDF) [file pmen.0000312.s010.pdf]

S8 Table. Responses from Music Therapy group feedback form.

Question	Result
1. Did you find this group helpful as a means of proactively managing stress and anxiety?	71% of the participants answered *yes*.
2. Please share any information you would like to share, such as examples of what was helpful/unhelpful, and if you learned anything through this experience about proactively managing your own wellbeing.	122 comments were identified. 70% of the comments related to music therapy being helpful. 30% of the comments related challenges. A thematic analysis was conducted revealing six subthemes related to helpfulness and five subthemes related to challenges (S7 File).
3. Would you participate in a music therapy group if it was offered at the University?	66% of the participants answered *yes.*
4. Would you attend the University's verbal based online therapy groups?	46% responded *yes*.
5. Please share any further information you would like to share.	• I don't think I'd care for online sessions.<br>• It was my first time participating in a study so it was interesting. Thank you.<br>• I am currently in verbal therapy as well, but I find I get different things out of that. That helps me find the root of some of my patterns, but music therapy helps me deal with everyday emotions.<br>• I have sought these out once and it was helpful when I did it. Also, it was online so maybe that made it seem easier to reach out for help.
