# Supplementary material for: Group music therapy for the proactive management of stress and anxiety
Source: PLOS Ment Health. 2025 Aug 14;2(8):e0000312. doi: 10.1371/journal.pmen.0000312 (PMC12798455; doi:10.1371/journal.pmen.0000312)
Supplement: S9 Table — Participants’ average scores from week 1 to week 6 in each of the four WHO-QOL categories. (PDF) [file pmen.0000312.s011.pdf]

S9 Table. World Health Organization Quality of Life BREF Scores, Week 1 and Week 6

	**Week 1** Music Therapy mean (SD)	**Week 6** Music Therapy mean (SD)	**Week 1** Control mean (SD)	**Week 6** Control mean (SD)
Physical health	66.2 (14.2)	67.7 (13.5)	70.9(15.1)	70.7 (13.2)
Psychological health	50.6 (17.7)	54.5 (9.1)	58.4 (17.5)	54.0 (8.9)
Social Relationships	60.6 (22.8)	64.7 (19.5)	67.5 (19)	68.2 (18.4)
Environmental Health	69.9 (12.2)	71.6 (12.1)	71.3 (13.6)	71.2 (13.9)
