# Supplementary material for: Group music therapy for the proactive management of stress and anxiety
Source: PLOS Ment Health. 2025 Aug 14;2(8):e0000312. doi: 10.1371/journal.pmen.0000312 (PMC12798455; doi:10.1371/journal.pmen.0000312)
Supplement: S1 File — (PDF) [file pmen.0000312.s012.pdf]

S1 File. Hair Collection Protocol, received by Drug Safety Laboratory, Robarts Research Institute, Western University

STEP 1: GATHER METERIALS FOR SAMPLE COLLECTION.
You will need: Sharp and clean scissors, Clear scotch tape, Hair clip (optional), Pen

STEP 2: LOCATE THE POSTERIOR VERTEX REGION OF THE SCALP.
This region of the head has the most consistent hair growth rate. Sampling from here will minimize the variation in sample measurements.

STEP 3: ISOLATE HAIR WITH HAIR CLIP (OR FINGERS) & CUT HORIZONTALLY WITH SCISSORS AS CLOSE TO SCLAP AS POSSIBLE.
Approx. 100 strands of hair are required, 1cm in length, 5mm in diameter (thickness of pencil eraser). Please ensure hair strands are aligned and secured with tape to the direction of the scalp and that the direction of the scalp is clearly indicated. Please use the collection form provided.

STEP 4:TAPE THE SCALP END OF THE HAIR TO THE HAIR SAMPLE COLLECTION FORM & FILL OUT STUDY ID, SUBJECT ID, & COLLECTION DATE.
Clear scotch tape works best. It is easily removed and leaves no residue on the hair. Fold the paper along the length of the hair & place in pre-addressed envelope to secure the sample.

Post the pre-addressed envelope after collecting. The pre-addressed envelope will be addressed to Drug Safety Laboratory, Robarts Research Institute, Western University, 1151 Richmond St N, London, On. Canada.
