# Supplementary material for: Group music therapy for the proactive management of stress and anxiety
Source: PLOS Ment Health. 2025 Aug 14;2(8):e0000312. doi: 10.1371/journal.pmen.0000312 (PMC12798455; doi:10.1371/journal.pmen.0000312)
Supplement: S3 File — (PDF) [file pmen.0000312.s014.pdf]

S3 File. Types of smart phones utilized by Welltory application for HRV data.

Smart phone	Data Collectors
Pixel 4a	x 1
iPhone 8	x 1
iPhone 10	x 1
iPhone 11	x 1
iPhone 12	x 4
iPhone 13 Pro	x 1
iPhone 13 ProMax	x 3
iPhone 14	x 1
iPhone 15	x 1
Samsung Galaxy S20	x 1
