# Supplementary material for: Group music therapy for the proactive management of stress and anxiety
Source: PLOS Ment Health. 2025 Aug 14;2(8):e0000312. doi: 10.1371/journal.pmen.0000312 (PMC12798455; doi:10.1371/journal.pmen.0000312)
Supplement: S4 File — (PDF) [file pmen.0000312.s015.pdf]

S4 File. Student Research Assistants. Group Music Therapy Sessions offered Tuesdays & Wednesdays 3-4 & 4:30-5:30

Student	Role
Erica Park	**Lead Research Assistant**
Fatima Arshad	**Lead Research Assistant**<br>Control Group
Tia Suomela	Data Collector
Shely Patel	Data Collector
Shruthi Raghuraman	Data Collector, Thematic Analysis
Navishka Brahmbhatt	Data Collector, Thematic Analysis
Melody Tjong	Data Collector
Gethmie Dep	Data Collector
Ayesha Gaba	Data Collector, Thematic Analysis
Jason Chung	Data Collector
Rayirth Sivakumar	Data Collector
McKenna ODonnell	Data Collector
Mansi Patel	Data Collector
Farhan Abdul Vaheed	Data Collector
Yatharth Utkarshkumar Dave	Data Collector
Josh Tony	Data Collector
Sana Alibhai	Data Collector (Group Lead)
Chloe Ko	Data Collector (Group Lead)
Cora House	Data Collector (Group Lead)
Matthew Wiebe	Data Collector (Group Lead)
Joyce Qiu	Data Collector (Group Lead)
Karina Kueviakoe	Data Collector, Scored Data, Organized Data, Categorized Ethnic origin comments
Carina Runco	Categorized Ethnic origin comments
