# Supplementary material for: Group music therapy for the proactive management of stress and anxiety
Source: PLOS Ment Health. 2025 Aug 14;2(8):e0000312. doi: 10.1371/journal.pmen.0000312 (PMC12798455; doi:10.1371/journal.pmen.0000312)
Supplement: S5 File — (PDF) [file pmen.0000312.s016.pdf]

S5 File. Structure of music therapy groups based upon: McMaster Wellness Centre Stress Less Group and Open Circle's Group Guidelines.

In keeping with the McMaster Student Wellness group "Stress Less" each group will explore the similarity and differences between stress, anxiety, and fear, identify stressors, explore stress physiology, discuss existing stress management strategies such as mindfulness.

*Group guidelines reviewed	Opening Quote & Ice Breaker	Facilitated reflection on a theme related to stress and anxiety	Closing activity & Quote

***Group Guidelines**
Adopted with permission from McMaster University Open Circle

- Give and receive welcome. People learn best in hospitable spaces. In this space we support each other's learning by giving and receiving hospitality.
- Respect: Listen to each other with openness and curiosity, being respectful of different ideas and opinions while open to learn from everyone.
- No fixing, saving, advising or correcting each other. This is one of the hardest guidelines for those of us who like to "help." But it is vital to welcoming the soul.
- Set aside reaction and judgment and turn towards wonder and compassionate inquiry. Ask yourself, "I wonder why they feel/think this way?" or "I wonder what my reaction teaches me about myself?" Set aside judgment to listen to others—and to yourself—more deeply.
- Speak your truth in ways that respect other people's truth. Our views of reality may differ, but speaking one's truth in this space does not mean interpreting, correcting or debating what others say. Speak from your center to the center of the group, using "I" statements, trusting people to do their own sifting and growth.
- Creating inclusive space for diversity. We work together to hold a safe space for all forms of diversity, including ethnicity, religion, gender identity and expression, sexual orientation, ability, and socio-economic status.
- Freedom to share or to pass. Be here with your listening as well as your speaking.
- Be present as fully as possible. Turn off phones and put away technology that is not being used for participation in the group.
- Trust and learn from the silence. Silence is a gift in our noisy world, and a way of knowing in itself. Treat silence as a member of the group. After someone has spoken, take time to reflect without immediately filling the space with words.
- Observe deep confidentiality. Safety is built when we can trust that our words and stories will remain with the people with whom we choose to share and are not repeated to others without our permission. Do not record any of the groups.
