# Supplementary material for: Group music therapy for the proactive management of stress and anxiety
Source: PLOS Ment Health. 2025 Aug 14;2(8):e0000312. doi: 10.1371/journal.pmen.0000312 (PMC12798455; doi:10.1371/journal.pmen.0000312)
Supplement: S6 File — (PDF) [file pmen.0000312.s017.pdf]

S6 File. Examples of specific Music Therapy interventions implemented.

- Musical check-in. Participants created a sound that described how they were feeling that day.
- Exploring different instruments, passing the various instruments around in a circle
- Participants sat in a circle facing the wall and engaging in improvisation and then turned around to face the group and improvise.
- Shared strategies on how to utilize mindfulness to help with stress and anxiety. Discussed importance of incorporating mindfulness as part of self-care in promoting wellness and healthy functioning.
- While playing "Rhapsody and Blue", each participant had a blank piece of paper and was asked to draw anything on their page based on what they heard, felt, pictured, when listening. After 2-3 minutes of listening, the music would pause and participants passed their sheet to the person to their right to continue to add to the drawing until each person's original paper returned to them. At the end, each participant held up their artwork.
- Music and breathing, box breathing technique
- Lyric analysis to the song "Stop this Train" by John Mayer. Participants listened to the music while following along to the lyrics on paper. Participants asked to reflect and think about the message of the song and if there were any specific lines in the piece that resonated with them.
- Utilized a template to the song "About Damn Time" by Lizzo and encouraged participants to create a song that is reflective of how they were feeling, values, etc. This provided the opportunity for participants to externalize their thoughts and emotions, tell their story, develop a sense of self, gain insight or clarify thoughts and feelings, choice and decision making.
- Collectively created a group music playlist which allowed participants to share some of their favourite songs.
- Participants reflected on songs that provide them with a sense of peace/grounding/reassurance/relief in the midst of stressful experiences.
- Split the group into two groups, participants selected an instrument to create a short piece with their group.
- Main themes included school stress and relationships (romantic and family)
- Music therapist sang and accompanied themself on guitar using songs chosen by the students (students played along with an instrument).
- Each week students were prompted to reflect on a quote accompanied by either recorded or live guitar music.
- Improvisation on themes related to stress (playing from stressed to relaxed).
- Collaborative song writing on stress using the song Stressed Out by 21 Pilots.
- Mantra writing/intention setting to music.
- Song share/discussion with drawing to music.
