# Supplementary material for: Group music therapy for the proactive management of stress and anxiety
Source: PLOS Ment Health. 2025 Aug 14;2(8):e0000312. doi: 10.1371/journal.pmen.0000312 (PMC12798455; doi:10.1371/journal.pmen.0000312)
Supplement: S7 File — (PDF) [file pmen.0000312.s018.pdf]

S7 File. Music therapy participants' individual responses to Question 2 of feedback form. Responses are not edited for typos etc., students were responding on a small tablet.

**2. Please add any information you would like to share, such as examples of what was helpful/unhelpful, and if you learned anything through this experience about proactively managing your own wellbeing.**
I was slightly stressed about the size of the hair sample taken from my head because I didn't know what to expect initially.
Very helpful group
Listening to music was helpful, as was progressive relaxation
That music is so versatile and you can use it in more than one way to cope with your stress or anxiety.
Though the last month I have had a lot of stuff going on in my life that have exacerbated some mental health issues, these sessions have helped me with tools to cope like the iso principle playlist as well as having a much deeper appreciation for music and what it can do for your brain and feelings. I find myself rereading and studying lyrics to specific songs over and over again as a means of therapy as well.
Anthony was absolutely fantastic! I knew that music was something that was therapeutic to me, but Anthony and this group really put into perspective to me how valuable music actually is to me as a stress relief resource. Between the Iso principles and just gaining an overall understanding of ways to implement music more purposefully as a stress relief tool in my life, I feel like I have a lot of good strategies and ideas to move forward with.
It was helpful having something to go to and feeling a sense of belonging
It was helpful in managing stress by making me actively think about how I subconsciously handle stressful situations and how to create better coping strategies
I really liked that the atmosphere was quiet and private really awesome. Cant think of any negatives
I think the question well reflected the focus on anxiety and student wellness.
I don't think I personally learned anything that I would use to proactively manage stress and anxiety but there were definitely things one could use such as progressive muscle relaxation if you notice you're beginning to feel stressed. I think a lot of the techniques are better for winding down from stress rather than preventing it. I find the "fun" aspect of it is most beneficial for me, seeing some friendly faces and having fun with music and conversing in a judgement free zone.
I found improvising with instruments particularly helpful and improved mindfulness. Music therapy was a place where I could feel secure and release any stressful obligations and worries. I enjoyed the reflection portion of music therapy and being able to talk and brainstorm with a small cohort of people. My least favourite part of music therapy was song writing. This part made me uncomfortable and did not improve mindfulness.
Sharing of thoughts and feelings towards songs, playing instruments wuth the therapist
Learning about the intentional therapy playlist was very helpful

I think it was helpful in that is provides prompts to actively think about mental wellbeing more in daily life; but it is hard to provide tangible help for immediate stressful situations, and a small group of strangers is not always very encouraging for mental health discussions
It was helpful to be given a place that kinda forced you to take time out of your day to be mindful. It was freeing to play with instruments like a littke kid again. Some parts felt almost 2vailable2t2 when in a group settimg, and felt like I I fully engage without feeking like others were watching. Afterwards I always felt more relaxed however, and I did enjoy hearing others experiences
It is great to get a chance to participant in the thearpy group. It is nice to share our thoughts and do not have to worry about our concerns.
I found the environment as the main source of relief, having positive relationships with the therapist and other participants was fun and offered me the chance the relax in an unfamiliar environment
I found the activities done in the group were really helpful, such as the drawing while listening to music and progressive muscle relaxation. I tried these at home too and they really helped.
It is pleasant at the moment but after that theres no difference
I felt content after the sessions.
During this process, I have learned to pay attention to the sources of my stressors. When I have many tasks to do for school as well as outside of school, I tend to ruminate about everything I have not done. This leads to more stress for the future. I have attempted to finish priority tasks and doing pieces of tasks overtime to manage my time effectively. Without the music therapy intervention, much of my stress management has been in my own hands. It has allowed me to practice different methods through trial and error.
Learning mindfulness and breathing techniques
The activities done during the sessions had a positive effects in the moment, but once the session was over it did not last.
I really enjoyed the progressive muscle relaxation, I can see myself using that as a way to be mindful og how im holding stress in my body.
I found that once I left the session I quickly returned to an anxious or stressed state. I also have my own strategies for coping with stress and anxiety that I feel work better for me. Some of the intervetions in the sessions felt a bit awkward or forced, like people did not really want to do them but participated out of obligation.
It was nice to be in a group and talk about stressors altogether
Making music with instruments was a great experience for reducing/distracting anxiety
There were always new things stressing me out each week so I was worried it wohld effect the study, and sometimes I also felt anxious to leave at the end to catch a bus which I felt may have effected my hrv
I foumd the muscle tensing and then relaxing exercise really helpful
The instrument parts, the feelings/thougths that were shared between us were helpful
Talking about stressors thru thenweek

Group therapy is just difficult for me
It was helpful however it would have been more helpful one on one.
The fact that it was in a group I help. People were less likely to share their thoughts because it was weird. Session also wasn't long enough.
It was a good exoerience but I feel that I would've gainee more if it waw one-on-one so I csn talk about things happening to me
I think that it was only mildly helpful during the sessions. Otherwise, I just forget about the coping strategies that were taught and cannot apply them in real life.
I feel as though any benefits in reducing stress through music therapy are minimal at best for me.
The sessions were helpful but I think they are only effective for a temporary period of time. I found the PMR at the end of the session relaxing though.
I appreciated the opportunity to participate overall however, I just feel as though group music therapy is not my thing. I may have felt differently if the sessions were one on one. I felt rather awkward at times participating in the activities with a group of people that I hardly know.
Not really
It was hard to take the tasks seriously
It is great to get the chance meet with the group and it is very fun being 3vailable3t in the group.
Using the instruments was very fun
Grouo setting just activated my social anxiety. Mindfulness excercises were goos to practice but private setting 3vailabl been more beneficial to me
I enjoyed when we drew pictures when listening to music but performing with instruements in front of everyone made be anxious
I learned to be present in the moment
I learned how music can be very helpful to maintain and imorove moods
I found this helped me to be present
I think in the final session, doing a group song and putting everyone on the spot for a solo kind of forced us to get out of our shell and participate, which was engaging.
Different methods to managr stress
I think this session mskes me feel relaexed and there are no right or wrong for music therapy
The therapist made the environment feelnsafe and casual with no pressure
I thought that the improvising with the instruments was too simple asnyou cant play any other note other than the bell that you have. Then again not everyome plays instruments
Being able to reflect my feelings was the best because I did not feel obligated to anything
I learned different techniques to use when managing stress to protect my mental wellbeing

I enjoyrd the drawing to music amd progressive muscle relaxation, both really relaxed me
I feel like just staying on campus longer getting work done waiting for this had is what hrloed mange my wellbeing
Made me feel less stress and gave some group emgagment
I liked sharing music we liked with the group amd drawimg to songs.
I found that listening to music was helpful but the progressive relaxation technique was unhelpful. I learned that it's important to stop and think about what you can control and not what you can't control.
I enjoyed learning how music can help with mental health and experimenting different instraments
Therapost made this space very safe
I feel like music therapy is a goof outlet for stress, I just I think it is a good outlet for me personally. I did not feel significant difference and I thought it was very time consuming,
While I did enjoy parts of the music therapy, I found that the group setting made it difficult to engage as it's very awkward with people you don't know really well. If music therapy was offered as a one on one therapy session, I think most people would be more comfortable opening up. However, I did find some of the techniques (especially the muscle relaxation) to be super helpful, and I will try incorporating them into my daily routine much more.
I really liked the muscle relaxation at the end of each session. I also liked listening to music together as that is something that has always helped me with stress. I did not love playing instruments because I found it a bit stressful, but I can see the value in it.
- Example of what was helpful: creating thoughtful, Iso-Principle playlists – to validate my current emotion and then using music purposefully to alter my state of mind gradually (travelling from an undesired (e.g., sadness/depression) to a desired/goal emotion (e.g., happiness))<br>- Example of what was helpful: improvising with instruments together, sometimes according to themes (e.g., confidence and connectedness) – enhanced sense of unity, was enjoyable to focus on my musical role in the group (wanted to make it sound good – allowed me to remain in the present moment and not let my thoughts interfere), etc.<br>- Something I have learned through this experience about proactively managing my well-being: to engage in the progressive muscle relaxation technique when I notice heightened stress/anxiety levels to ground myself, feel more secure and reframe my thoughts (from music therapy experience in a GROUP, I may consider engaging in this technique with friends/family rather than alone to achieve the same successful results – helps me to focus more by feeling motivated in the presence of others)
I will really miss these sessions, if they became available again at McMaster I would really like to be informed and take part.
I think music therapy is a powerful proactive measure and I wish it could be made 4vailable to younger populations and more routinely
Perhaps the group aspecf made it feel less personal. If it was one on one I believe I would have benefitted more
I would participate if it was one on one instead of a group.

I think I great practice and the instructor was very talented, though movimg forward Id prefer somerhing like a band over therapy. Even so, hearing the opinions of everyone in the session was insightful.
I think that having groups with a specific healthcare goal in common would be helpful.
Overall, I thoroughly enjoyed participating in weekly group music therapy sessions! Rather than feeling like an endless, tedious chore or task to complete every Wednesday at 3:00pm, these felt like a fun, educational break from the business and stresses of life. The psychotherapist/music therapist fostered a safe, supportive, and enjoyable environment for myself and the rest of my group members! Through engaging in a progressive muscle relaxation technique every session, analyzing song lyrics, improvising together with instruments according to positive themes, and creating brief playlists that allow you to travel from an undesired to a desired emotion or to complete a daily task, such as fully waking up in the morning, I have better learned how to manage my stress/anxiety levels by incorporating different mediums of music into my life! To future HUMBEHV 2AP3 students: I highly recommend partaking in this study if you have the opportunity to do so.
